# Evaluation of a new venous catheter blood draw device and its impact on specimen hemolysis rates

**DOI:** 10.1016/j.plabm.2018.01.002

**Published:** 2018-01-04

**Authors:** Ruth Natali, Cara Wand, Kelly Doyle, Jaime H. Noguez

**Affiliations:** a Department of Pathology, University Hospitals Cleveland Medical Center, 11100 Euclid Ave, PATH 5077, Cleveland, OH, USA; b Department of Pathology, Dixie Regional Medical Center, St. George, UT, USA; c Central Laboratory, Intermountain Healthcare, Murray, UT, USA; d Department of Pathology, Primary Children's Hospital, Salt Lake City, UT, USA; e Department of Pathology, University of Utah, Salt Lake City, UT, USA; f Department of Pathology, Case Western Reserve University, Cleveland, OH, USA

**Keywords:** Venipuncture, Hemolysis, PIVO™, Peripheral IV blood collection, Patient experience, Blood collection

## Abstract

**Objectives:**

Blood collections from peripheral intravenous catheters offer several benefits to patients, including reduced needle punctures and patient discomfort, but they risk reducing the quality of blood specimens analyzed by the laboratory. In an effort to balance analytical quality of test results with patient-centered care initiatives, a needle-less blood collection device called PIVO™ was evaluated at two institutions. The primary objective of this study was to assess the ability of the PIVO™ device to provide high-quality blood specimens for laboratory testing compared to current blood collection methods.

**Methods:**

Blood specimens drawn using the PIVO™ device were prospectively flagged. A retrospective review was performed comparing the degree and rate of hemolysis for PIVO™ blood collections to both concurrent and historical hemolysis rates for other collection methods.

**Results:**

Approximately 7600 PIVO™ blood draws were performed across the two institutions. The hemolysis rates of samples collected with PIVO™ were evaluated using 2380 flagged collections, containing approximately 1200 test orders requiring hemolysis index measurements. The hemolysis rate of PIVO™-flagged samples (1.8%) was statistically superior to the venipuncture and central line blood collection methods (3.3%), reducing the risk of hemolysis during a venous blood draw by 39%.

**Conclusions:**

PIVO™ collections facilitated improvement in the rate and degree of sample hemolysis when compared to venipuncture and central line blood collections. These findings suggest that PIVO™ is capable of delivering samples that are superior to current blood collection methods in terms of hemolysis rate as well as reducing the number of invasive venipunctures required for laboratory testing.

## 1 Introduction

Hemolysis, defined as the breakdown of red blood cells and the release of hemoglobin and intracellular contents into the plasma, is a frequent occurrence in blood samples submitted to clinical laboratories for testing. The estimated prevalence of hemolyzed specimens is approximately 3% of routine samples and they make up approximately 60% of specimens classified as unsuitable for analysis [1]. Higher rates of hemolysis are typically seen in the acute care setting, such as the emergency department and intensive care units, with some literature reports as high as 32% [1], [2]. Sample hemolysis is a patient safety concern because it can lead to inaccurate laboratory results, the need for specimen recollection, and potential delays in diagnosis and treatment. There are several laboratory tests that can be impacted by hemolysis with varying levels of interference observed based on the degree of hemolysis and the specificity of the method being used. The risks associated with analyzing hemolyzed specimens have been recognized and efforts made toward creating more robust assays and facilitating consistent high quality specimen collections.

Previous research has extensively explored the causes of hemolysis including patient inherent factors, collection techniques, collection devices, and specimen transport. Collection from a peripheral IV is one of the most commonly cited causes of hemolysis and is associated with decreased specimen integrity as compared to venipuncture due to increased mechanical shearing of the red blood cells [3], [4], [5]. In fact, one study found that specimens drawn though an IV catheter were more than 3 times as likely to be hemolyzed than those drawn by venipuncture [4]. Not surprisingly, the highest rates of hemolyzed specimens are observed in acute care settings where collections through peripheral IV catheters are most common [1]. The patient populations in these units often consist of individuals in which double needle punctures are avoided to improve efficiency and patient experience such as pediatric patients or adults with difficult venous access, presence of bleeding disorders, or the need for serial testing [6]. Efforts to reduce hemolysis rates in these settings have largely been focused on standardization of blood collection practices, staff training initiatives to improve technique, and in recent years the development of new blood collection devices.

**Fig. 1:**
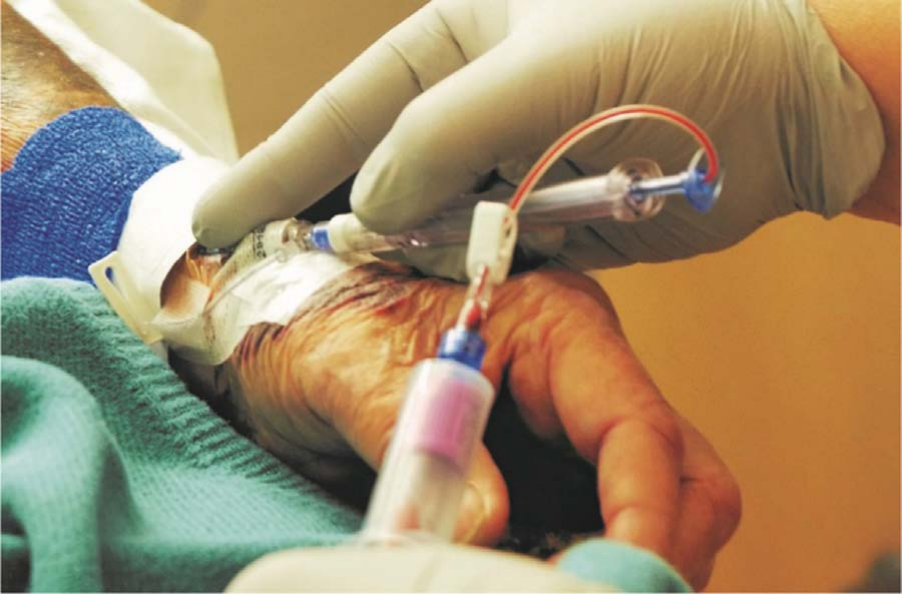
PIVO™ device collecting blood.

In 2016, the clinical laboratories of Dixie Regional Medical Center (DRMC, a 130-bed level II trauma center) and University Hospitals Cleveland Medical Center (UHCMC, a 1100-bed level I trauma center) each embarked on an evaluation of a new needle-free blood collection technology. PIVO™ (Velano Vascular Inc.) is a single-use, FDA-cleared blood collection device that connects to the hub of a peripheral IV catheter (PIVC) extension set and advances a polymer cannula through the PIVC into the vein to collect a blood specimen (Fig. 1). The goal of this evaluation was to assess if the needle-free blood collection method could deliver blood specimens of equivalent integrity to venipuncture. The hemolysis rate from nearly 1200 PIVO™ blood collections across both institutions was compared to concurrent and historical venipuncture hemolysis rates using the specimen integrity check feature of the automated chemistry analyzers. This large, statistically significant sample set was taken from real clinical usage of the product at these institutions and used to validate PIVO™ as a beneficial option to both the laboratory and patients.

## 2 Materials and methods

### 2.1 Blood collection materials

BD Vacutainer Safety-Lok Blood Collection Sets (Becton Dickinson, Franklin Lakes, NJ) with pre-attached holders were used for venipuncture at DRMC and UHCMC. DRMC also utilized BD Eclipse needles with holders and UHCMC used Greiner Bio-One Vacuette tubes (Greiner Bio- One, Monroe, NC). Central line collections used the same holders as the venipunctures at each hospital. The PIVO™ blood collection device was used for blood draws from a peripheral IV catheter. Prior to the PIVO™ collection, the IV catheter was flushed with 5 mL normal saline. The PIVO™ device was attached to the needle-less valve and actuated through the IV catheter into the blood stream. Standard vacuum tubes or a syringe were used at the back end of the device to collect blood samples. The blood collections were typically performed with a tourniquet above the PIVC. A discard volume of 1 mL was collected through PIVO™ prior to the required specimen collection based on research by Baker [7]. After the PIVO™ collection the device was retracted, removed, and disposed of. The IV catheter was again flushed with 5 mL normal saline.

### 2.2 Laboratory instrumentation and indices

Patient testing was performed on the Siemens Dimension Vista at UHCMC prior to June 25, 2016 and on the Beckman Coulter AU5800 thereafter. The Abbott Architect ci8200 was used for patient testing at DRMC. Specimen integrity was checked by the semi-quantitative, spectrophotometric assessment of hemolysis in human serum and plasma on these automated chemistry analyzers. Only specimens with orders for blood chemistry tests known to be affected by hemolysis, as described in the assay manufacturer package insert, were subjected to automated specimen integrity checks. A hemolyzed specimen was defined as having a free hemoglobin concentration ≥50 mg/dL, which is known to confer a detectable pink to red hue to serum/plasma that may affect the accuracy of some laboratory test results [8], [9]. This level of free hemoglobin corresponds to a hemolysis index of 4 on the Dimension Vista and 1 on both the Beckman AU and Abbott Architect. At both DRMC and UHCMC the rate of hemolysis was defined as the percentage of specimens run on the chemistry analyzers that flagged as hemolyzed relative to all specimens with serum indices measurements.

### 2.3 PIVO™ utilization

**Fig. 2:**
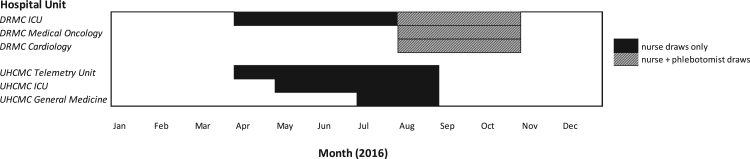
PIVO™ Utilization at UHCMC and DRMC in 2016.

The PIVO™ device was introduced at DRMC on one intensive care unit in April 2016 and was initially used by nurses to collect labs from patients with a PIV catheter. In August 2016, the device usage was expanded to trained phlebotomy staff for use on this unit as well as a cardiovascular unit and medical/oncology unit. The evaluation period lasted through October 2016. The device was also introduced at UHCMC in April 2016 and evaluated through August 2016. Blood collections were expanded progressively to three units beginning with a telemetry floor, growing to include an intensive care unit, and finally including a general medical floor (Fig. 2). At UHCMC, PIVO™ was used by nurses only, which changed the usual blood collection workflow for laboratory testing from phlebotomists to nurses in two of the units. In the intensive care units nurses routinely collect blood samples for laboratory testing whereas on the telemetry and medical units nurses were newly tasked with blood collections typically performed by laboratory phlebotomy staff.

### 2.4 Data collection

As part of internal quality measures performed by the laboratory, this data analysis was exempt from institutional review board review. An effort was made to prospectively flag the blood samples collected with the PIVO™ device such that a retrospective review could be performed in aggregate. At both institutions, cards were used to designate samples as being collected by PIVO™. When a sample was collected, the practitioner was instructed to place a colored card inside the biohazard bag containing the blood samples and deliver it to the lab in accordance with normal procedures. Practitioners were strongly encouraged to flag all samples collected with PIVO™, however, there was no mechanism to ensure that all samples collected with PIVO™ were identified as such.

All samples sent to the laboratory, including PIVO™ and non-PIVO™ specimens, were run using routine processes as described above. Hemolysis flags were appended to test results when the hemolysis index corresponding to a free hemoglobin concentration of ≥ 50 mg/dL was triggered for blood chemistry test samples requiring an automated specimen integrity check. Data on hemolysis rates was extracted from the laboratory information system for all samples processed by the lab during the evaluation period. At UHCMC, data extraction was limited to the floor on which the PIVO™ device was evaluated. At DRMC, hemolysis rates from all inpatient blood collection data was pulled because use of the PIVO™ device was eventually transitioned for use across the entire hospital. Historical hemolysis data was also extracted for comparison. At DRMC inpatient blood collection data from the period of October 2015 through March 2016 provided a 6-month historical comparison. At UHCMC data from evaluation units from September 2015 through March 2016 was used as a historical comparison.

### 2.5 Data analysis

Statistical analysis was performed by an independent group, Biomedical Statistical Consulting, LLC. For each institution, an exact binomial confidence interval for the difference in hemolysis rates between PIVO™-flagged samples and all other samples was calculated, as well as a chi-square p-value on the likelihood that the difference in hemolysis rates was greater than zero. These particular statistical tests were chosen because of their ability to detect differences in populations with potentially high standard deviations. Two comparisons were performed: 1) the hemolysis rate of PIVO™-flagged samples during the pilot studies compared to the historical hemolysis rate for all methods of blood collection before the pilot studies began, and 2) the hemolysis rate of PIVO™-flagged samples compared to all other draws performed in the evaluation period. It should be noted that PIVO™-flagged samples were all collected with the PIVO™ device and all historical collections were obtained by venipuncture or through a catheter without PIVO™. However, the “all other draws” performed in the evaluation period includes venipunctures, line collections, and those PIVO™ collected samples which were not flagged with a tracking card. Prior to pooling the results across the two institutions, the Breslow-Day statistic was used to assess whether the relative hemolysis rates of PIVO™ and non- PIVO™ collected samples were consistent across institutions. Once it was established that the group differences did not vary by institution, the Cochran-Mantel-Haenszel statistic was performed to aggregate the relative risk of hemolysis from PIVO and non-PIVO draws across institutions while adjusting for possible confounding effects at each institution.

## 3 Results

During the evaluation period, an estimated 4100 blood collections were completed with PIVO™ at DRMC and approximately 3500 blood collections were performed with PIVO™ at UHCMC. Imperfect compliance with the practice of identifying PIVO™ blood collections resulted in only 1999 PIVO™ identified collections from DRMC and 381 PIVO™ identified collections from UHCMC. These data indicated an estimated PIVO™ draw card compliance rate of 48% at DRMC and 11% at UHCMC. Of the PIVO™-identified collections, DRMC had 897 and UHCMC had 286 samples on which hemolysis data was reported by the analyzers.

At both DRMC and UHCMC, the hemolysis rate from the PIVO™-flagged samples was lower than both the historical and concurrent rates from other blood collection methods (Table 1). Due to the greater difference in hemolysis rates, the comparison at UHCMC showed PIVO™ to be statistically superior to other concurrent collection methods even though the sample size was smaller. At DRMC, the reduction in hemolysis rate was not statistically significant. No significant differences were found in hemolysis risk by site using the Breslow-Day test (p > 0.10) enabling an aggregate data analysis to be performed. Under the aggregate relative risk analysis, the hemolysis rate of PIVO™-flagged samples was shown to be statistically superior to the hemolysis rate of other blood collection methods under both (historical and concurrent) comparisons (Table 1).

**Table 1:** DRMC and UHMC hemolysis rates pre-PIVO™ pilot and during pilot.

	Historical all blood collections	Hemolysis rate	All other collections during evaluation	Hemolysis rate	PIVO-flagged collections	Hemolysis rate	Difference to historical	Difference to concurrent
DRMC	22,594	2.3% (510)	23,877	2.4°% (567)	897	1.6°% (14)	−0.7%; CI(−1.5% to 0.1%), p−0.17	−0.8%; CI(−1.6% to 0.0%), p−0.11
UHCMC	19,215	4.3% (824)	12,709	5.2°% (658)	286	2.4°% (7)	−1.8%; CI(−3.1% to 0.0%), p−0.13	−2.7%; CI(−4.6% to −1.0%), p−0.04
Aggregate							−1.4%; CI(−2.2% to −0.6%), p−0.01	−1.6%; CI(−2.4% to −0.8%), p−0.00

Data on the degree of hemolysis was also obtained. None of the 14 hemolyzed PIVO™-tagged specimens at DRMC had a hemolysis index >100 mg/dL whereas hemolysis was greater than >100 mg/dL in 32% of hemolyzed specimens that were non- PIVO™ tagged. At UHCMC, 2 (29%) of the 7 hemolyzed PIVO™-tagged specimens showed hemolysis of greater than 100 mg/dL of hemoglobin, which was equivalent to the percentage of non- PIVO™ hemolyzed samples showing greater than 100 mg/dL of hemoglobin (29%).

## 4 Discussion

Laboratory medicine is constantly improving by utilizing new technologies and building from the best available evidence. A consensus of evidence in the literature suggests that while PIVC blood collections are understood to offer benefits to patients, they risk reducing the quality of blood specimens analyzed by the laboratory. The PIVO™ device was evaluated at DRMC and UHCMC because it offered an opportunity to improve the patient experience without the loss of sample integrity common to peripheral IV collections. We quickly recognized the potential widespread impact of the device considering that an estimated 60–90% of hospitalized patients in the United States require an IV catheter during their stay [10].

Approximately 7600 PIVO™ blood draws were performed across these two institutions. The hemolysis rates of samples collected with PIVO™ were measured using the 2380 flagged collections which contained approximately 1200 samples with a reported hemolysis index. The hemolysis rate of PIVO™-collected samples compared favorably to the rates from other blood collections done prior to and during the PIVO™ pilot study. Differences in blood draw practices, the ratio of IV line draws to other collection methods, the acuity of patient illness, and patient populations are among the myriad of potential causes of the different baseline hemolysis rates observed between institutions in this study. Regardless of these differences, the trend of decreased sample hemolysis with the use of the PIVO™ device was observed at both hospitals. In fact, the aggregate data analysis suggests that the PIVO™ device reduced the risk of hemolysis during a venous blood draw by 39% (Risk ratio .61; CI .40–.94) compared to the other collection methods being evaluated. The degree of hemolysis found upon detection, however; only decreased in PIVO™-drawn specimens at DRMC and was equivalent at UHCMC regardless of the blood collection method used.

A limitation of this quality study is the imperfect tracking of PIVO™ collected specimens. PIVO™-flagged specimens can therefore only be imperfectly compared to all other draws during the pilot, which also includes a portion of PIVO™ collected specimens submitted without tracking cards. However, the consistency in findings between historical error rates and concurrent errors rates substantiates the results. Overall, these findings suggest that PIVO™ is capable of delivering samples that are superior to current blood collection methods in terms of hemolysis rates.

The lower rates of hemolysis obtained with PIVO™ collections may possibly be explained by the fluid path of the blood as it is being drawn through the catheter. *Ex-vivo* hemolysis is understood to be caused by disruption of cells either during the collection process or in specimen handling following collection. While the suction force on cells during aspiration alone can cause some lysis, the turbulence of the blood pathway is thought to be a main driver of cell breakage. Venipuncture collections draw cells through a straight fluid path into the collection device. In contrast, cells drawn through a peripheral IV catheter or central catheter and its attached needleless connector follow a particularly tortuous path which can cause higher levels of hemolysis due to mechanical stress [3]. By temporarily threading the PIVO™ device through the lumen of a PIV catheter, PIVO™ blood collections mimic the straight fluid path of a venipuncture, possibly explaining the lower hemolysis rate. A previous study comparing PIVO™ blood collection to venipuncture demonstrated a zero percent hemolysis rate for both methods [11]. However, PIVO™ blood draws in real clinical use, like venipuncture collections, are open to handling errors such as pulling too vigorously when drawing with a syringe or re-centrifugation of a blood tube that did not achieve a clearly defined separation between the blood cells and serum/plasma on the initial spin.

Following the evaluation it was noted that use of the PIVO™ device positively impacted some aspects of the laboratory blood collection workflow. In both hospitals, a 15–30 min in-service was provided to all users to ensure proper use of the device. After a few draws most users were comfortable with the technique and felt that they were able to complete blood draws in the same amount of time as without the device, if not less. At UHCMC, where the PIVO™ device was used solely by the nursing staff, it was observed that the phlebotomy staff did not spend as much time, if any, on the PIVO™ pilot floors allowing them to complete their rounds more quickly. This was particularly helpful for such a large institution with lean phlebotomy staffing. Additionally, the ability to obtain blood samples more easily on patients described as a “hard stick” was a recognized benefit of PIVO™ use. At DRMC, where the PIVO™ devices were used by both nursing and laboratory staff, the phlebotomists expressed that they enjoyed being part of the trial because patients appreciated not being subjected to traditional venipuncture and described the patient-phlebotomist interaction as a more positive experience. A small subset of patients surveyed validated these comments, suggesting that patients look favorably upon the needle-free collection method. Unfortunately, no conclusions can be made at this time regarding the impact of the PIVO™ device on hemolysis rates when used by nursing versus phlebotomy staff due to the small number of hemolyzed specimens observed at both institutions.

These pilot studies demonstrate that the PIVO™ device has potential to simultaneously improve the quality of specimens received in the laboratory from acute care units, the patient blood collection experience, and phlebotomy workflow. The current device only works through 22 gauge IVs and larger and is therefore not yet applicable to neonates. Further research and development will hopefully allow the benefits of the device to be realized by the pediatric population in the near future as well as the possibility for a reduced discard volume.
